# Astragaloside IV Alleviates Infarction Induced Cardiomyocyte Injury by Improving Mitochondrial Morphology and Function

**DOI:** 10.3389/fcvm.2022.810541

**Published:** 2022-02-21

**Authors:** Wen Zhang, Ling Zhang, Huifen Zhou, Chang Li, Chongyu Shao, Yu He, Jiehong Yang, Haitong Wan

**Affiliations:** ^1^College of Life Science, Zhejiang Chinese Medical University, Hangzhou, China; ^2^College of Pharmaceutical Science, Zhejiang Chinese Medical University, Hangzhou, China; ^3^College of Basic Medical Sciences, Zhejiang Chinese Medical University, Hangzhou, China

**Keywords:** astragaloside IV, myocardial infarction, mitochondrial function, silent information regulator 3, dynamin-related protein 1

## Abstract

The protective effect of astragaloside IV (AS-IV) on myocardial injury after myocardial infarction has been reported. However, the underlying mechanism is still largely unknown. We established a myocardial infarction model in C57BL/6 mice and injected intraperitoneally with 10 mg/kg/d AS-IV for 4 weeks. The cardiac function, myocardial fibrosis, and angiogenesis were investigated by echocardiography, Masson's trichrome staining, and CD31 and smooth muscle actin staining, respectively. Cardiac mitochondrial morphology was visualized by transmission electron microscopy. Cardiac function, infarct size, vascular distribution, and mitochondrial morphology were significantly better in AS-IV-treated mice than in the myocardial infarction model mice. *In vitro*, a hypoxia-induced H9c2 cell model was established to observe cellular apoptosis and mitochondrial function. H9c2 cells transfected with silent information regulator 3 (Sirt3) targeting siRNA were assayed for Sirt3 expression and activity. Sirt3 silencing eliminated the beneficial effects of AS-IV and abrogated the inhibitory effect of AS-IV on mitochondrial division. These results suggest that AS-IV protects cardiomyocytes from hypoxic injury by maintaining mitochondrial homeostasis in a Sirt3-dependent manner.

## Introduction

Myocardial infarction (MI) refers to the ischemic necrosis of the myocardium. In coronary artery disease, the blood flow of the coronary artery is sharply reduced or interrupted, resulting in serious and lasting acute ischemia in the corresponding myocardium. This eventually leads to the ischemic necrosis of the myocardium and seriously endangers patient health (1). Therapeutic angiogenesis aims at treating ischemic diseases by generating new blood vessels from existing vasculature (2). Therapeutic angiogenesis has been widely examined for treatment of many human diseases, such as wound healing and organ repair and regeneration (3). The pathogenesis of myocardial injury mainly includes energy metabolism disorder (4), free radical injury (5), calcium overload (6), and inflammatory response (7), which are related to mitochondria.

In recent years, the role of mitochondria in the process of myocardial ischemia has attracted extensive attention. Protecting the structure and function of mitochondria and enhancing the ischemic tolerance of mitochondria has become an important strategy to prevent and treat myocardial injury (8). Silent information regulator 3 (Sirt3) is one of seven members of the mammalian sirtuin family. Sirt3 balances the redox state of cells by the regulating metabolism to stabilize cell energy and regulate enzyme activity (9). The fusion of the mitochondrial inner membrane is mediated by optic atrophy protein-1 (Opa1), which is located on the inner membrane (10). Dynamin-related protein 1 (Drp1) is a major protein that mediates mitochondrial division (11). Evidence suggests that mitochondria constantly fuse and divide for self-renewal, changing their own morphology, interconnecting, and maintaining their own steady state. An unbalanced process of mitochondrial fusion and division will affect the morphology and function of the mitochondria; this mitochondrial dysfunction leads to cardiac dysfunction (12, 13). In a study on mitochondrial dynamics, Sirt3 enhanced the activity of Opa1 and inhibited the activity of Drp1 by modifying, regulating the fusion and cleavage of mitochondria, and affecting the functional state of mitochondria, so as to maintain the steady state of mitochondria in myocardial injury (14, 15).

*Astragalus membranaceus* is a traditional Chinese herbal medicine that is thought to promote health. Astragaloside IV (AS-IV) is the main active component of *Astragalus*. AS-IV has a certain effect on inflammation by inhibiting the pro-inflammatory factor high mobility group protein 1, which has a potential effect on regulatory T cells (16). Studies have suggested that AS-IV has a protective effect on brain injury (17), lung injury (18), kidney injury (19), and heart injury (20) caused by ischemia-reperfusion. AS-IV showed anti-inflammatory activity by inhibiting NF-κB pathway (21). Previous studies have confirmed that AS-IV has a positive effect on MI (22–24). The potential mechanism of AS-IV in the treatment of MI might involve the downregulation of CaSR expression, upregulation of ERK1/2 phosphorylation (25), inhibition of nuclear NF-κB p65 subunit translocation to the nucleus (26), downregulation of TLR4/NF-KB expression (27), upregulation of HIF-1A expression (28), changes in Ca^2+^-ATPase activity (29), improvement in myocardial energy metabolism (30), and regulation of the mitochondrial apoptosis signaling pathway (31). However, the mechanisms by which AS-IV regulates mitochondrial homeostasis in the treatment of MI remain unknown. We hypothesized that AS-IV ameliorates MI by regulating key mitochondrial proteins (Sirt3, Opa1, and Drp1). This study mainly focused on the role mitochondrial homeostasis in the protection of cardiomyocytes by AS-IV.

## Materials and Methods

### Drugs and Reagents

AS-IV (batch number: 140913) was obtained from Weikeqi Biotechnology (Sichuan, China). Betloc tablets (batch number: 2010038) were purchased from AstraZeneca (Wuxi, China). Cell Counting Kit-8 (CCK-8, CAS Number: ZP328-3) was purchased from Zomanbio (Beijing, China). ATP Assay kit (CAS Number: S0026), mitochondrial membrane potential assay kit with JC-1 (CAS Number: C2006), and reactive oxygen species assay kit (CAS Number: S0033S) were purchased from Beyotime Biotechnology (Shanghai, China). RiboFECT transfection kit (CAS Number: C10511-1) was obtained from Ribo Biotechnology (Guangzhou, China). Mitochondrial isolation kit (Lot#019M4159V) was purchased from Sigma (St. Louis, USA). Troponin I antibody (CAS Number: ab209809), CD31 antibody (CAS Number: ab222783), α-smooth muscle actin (α-SMA) antibody (CAS Number: ab124964), BAX antibody (CAS Number: ab32503), Bcl2 antibody (CAS Number: ab196495), cytochrome C antibody (CAS Number: ab133504), Sirt3 antibody (CAS Number: ab246522), Opa1 antibody (CAS Number: ab42364), VDAC1 antibody (CAS Number: ab15895), and Drp1 antibody (CAS Number: ab184247) were obtained from Abcam (Cambridge, MA, USA).

### MI Model and Treatment

The animal experiments conducted in this study strictly complied with the National Institutes of Health Guide for Care and Use of Laboratory Animals (32). The animal research protocol was approved by the Institutional Animal Care and Use Committee of the Laboratory Animal Research Center of Zhejiang Chinese Medical [License No. SYXK (Zhe)2018–0012].

Eight-week-old healthy male C57BL/6 mice (SPF quality standard) were purchased from Shanghai Slaker Company and fed in an SPF quality standard animal room (temperature 23 ± 2°C, humidity 50–70%, 12 h light/dark cycle, and water *ad libitum* for 1 week).

For MI induction, 40 male C57BL/6 mice were anesthetized with 0.3% pentobarbital at the dosage of 50 mg/kg followed by endotracheal intubation, supine fixation, and ethanol disinfection of the incision fur. Along the line between the axilla and lower sternum, a 1.5 cm incision was made in the third and fourth intercostal spaces of the heart, and the thoracic wall muscle tissue was obtusely separated. The left main descending branch of the coronary artery was ligated with no. 6-0 silk line. After the cardiac apex turned gray, the MI model was considered to be successfully established. In the normal group, the incision was made and the left anterior descending coronary artery was separated without ligation (33).

The model mice were randomly divided into four groups, the normal group, the model group (*n* = 10), in which the mice were orally administered distilled saline; the AS-IV treatment group (*n* = 10), in which the mice were injected intraperitoneally with 10 mg/kg AS-IV (34) for 4 weeks; and the positive control group (*n* = 10), in which the mice were orally administered 18 mg/kg Betloc (Bet) (35) for 4 weeks. Bet is a β1-selective blocker that reduces blood pressure and heart rate, increases ventricular diastolic time, and improves cardiac function. Ibanez et al. (36–38) have demonstrated the important role of bet in the treatment of patients with MI.

### Echocardiography

Cardiac function was non-invasively monitored using a Vevo1100 system with a 15MHz probe. The ejection fraction (EF) and fractional shortening (FS) were the calculated parameters.

### Hematoxylin and Eosin Staining

The apical tissues of the mice were fixed with 4% paraformaldehyde at room temperature, embedded in paraffin, and sectioned. Prior to analysis, the paraffin sections were dewaxed in water, stained with H&E, dehydration sealed, observed under microscope, and imaged.

### Masson Staining

The paraffin sections were dewaxed in water, stained with iron hematoxylin, Ponceau red, acid fuchsin, phosphomolybdic acid, and aniline blue, dehydration sealed, observed under microscope, imaged, and analyzed.

### Immunofluorescence

After dehydrating the paraffin sections, antigen repair was performed, and the sections were blocked with 5% goat serum at room temperature for 30 min. Subsequently, the serum was discarded, and primary antibody was added (troponin I 1:300 dilution, CD31 1:100 dilution, α-SMA 1:300 dilution) followed by incubation at 4°C overnight. On the next day, the sections were reheated at room temperature for 1 h, rinsed three times with phosphate-buffered saline (PBS), dropped with fluorescent secondary antibody (1:300 dilution), and incubated at room temperature for 1 h in the dark. After rinsing three times with PBS, DAPI was added, and the sample was observed under a fluorescence microscope.

### Transmission Electron Microscopy

The apical tissues of mice were cut into 1-mm^3^, fixed in 2.5% glutaraldehyde, rinsed with PBS, refixed with 1% Qian acid, dehydrated stepwise using acetone, soaked in 812 embedding agent, embedded, and solidified. Ultrathin sections (70 nm) were double stained with uranium acetate and lead citrate. The structural changes of the mitochondria were observed under a transmission electron microscope.

### Western Blotting Analysis

Western blotting was used to detect the expressions of Sirt3, Drp1, and p-Drp1. The apical of the mice hearts was used for protein extraction and Western blot analysis. Cardiac tissues were fully ground in liquid nitrogen and lysed using RIPA buffer with 10% protease inhibitor cocktail and 10% phosphatase inhibitor. Protein concentrations were determined by Pierce BCA protein assay kit. The total protein was analyzed by electrophoresis for 2 h. The membrane was turned at a constant pressure of 100 V for 2 h followed by blocking with 5% bovine serum albumin (BSA) for 1 h, incubating overnight with primary antibody (1:1,000) at 4°C, and incubating with secondary antibody (1:5,000) at room temperature for 1 h. The bands were then visualized using an enhanced ECL reagent (Bio-Rad, USA). The levels of each protein relative to β-actin were quantified using Image J software (National Institutes of Health, Bethesda, MD, USA).

### Cell Culture and Hypoxia Condition

H9c2 cardiomyocytes were cultured in Dulbecco's modified eagle medium (DMEM) containing 10% fetal bovine serum (FBS) and placed in a cell incubator containing 5% CO_2_ at 37°C (39). FBS was inactivated before use. Penicillin (100 u · ML^−1^) and streptomycin (100 u · ML^−1^) were added to the culture medium to prevent bacterial infection. The culture medium was changed every 2 days and subculturing was conducted when the cell density reached 80–90% of the Petri dish by area. The experimental cells were randomly divided into the three groups (normal, model, and AS-IV groups).

Based on previous reports, the conditions for establishing the model were 4 h of hypoxia (40). H9c2 cells resuspended. When cells grew to ~80% of the Petri dish by area, the medium was removed, and the cells were washed with PBS. Serum-free and sugar-free DMEM was then added to the cells, which were placed in a self-made anaerobic tank, sealed, and cultured for 4 h to make the cells hypoxia.

### Transfection

The three siRNAs targeting Sirt3, negative control siRNA, and transfection kit were obtained from Ribo Biotechnology Co., Ltd. (Guangzhou, China). Transfection reagent was added to the medium to facilitate transfection, following the manufacturer's instructions. Twenty-four hours later, the efficiency of Sirt3 silencing was measured *via* western blotting. Sirt3-siRNA-3 exhibited the best interference efficiency and labeled the treated cells as the Sirt3-siRNA group.

The H9c2 cells were randomly divided into 5 groups: (1) Normal; (2) Model + NC-siRNA; (3) Model + Sirt3-siRNA; (4) AS-IV + Sirt3-siRNA; (5) AS-IV.

### Cell Viability Assay

Logarithmic H9c2 cells were inoculated in 96-well-plates at a density of 0.6 × 10^5^ cells/well in 100 μL medium under 5% CO_2_ at 37°C. Subsequently, different concentrations of AS-IV (0.005, 0.01, 0.05, 0.1, 0.5, 1, 5, 10, 20, 50, and 100 μmol/mL) were added to establish the model according to the above method. Cell viability was estimated by CCK-8 assay. The absorbance (optical density, OD) of each well was measured at 450 nm using a microplate reader (Molecular Devices Spec-tra MAX Plus 384, MD, USA). The same amount of CCK-8 solution was added to the cell-free culture medium. The absorbance was measured according to the same method used for the control, and the cell survival rate was calculated as follows: cell survival rate (%) = (OD value of experimental hole – OD value of blank hole) ÷ (OD value of control hole – OD value of blank hole) ×100%. The optimal concentration of drug was 1 μmol/mL, which was selected for the subsequent mechanistic study.

### Measurement of ATP Level

The intracellular ATP content was analyzed using an ATP assay kit according to the manufacturer's instructions. After corresponding treatments, 100 μL of ATP detection working solution containing 10 μL of the supernatant sample was added to a white 96-well-plate for luminescence analysis using a microplate reader. A standard curve was drawn using ATP standard solution to determine the concentration of ATP.

### Mitochondrial Membrane Potential

H9c2 cardiomyocytes in the logarithmic growth stage (6 × 10^4^ cells/well) were inoculated in a 10-mm laser confocal culture dish. The cells were cultured according to the method in Section Western Blotting Analysis, and cleaned twice with PBS, after adding 100 μL of serum-free DMEM and 100 μL of JC-1 solution to each well, the cells were incubated at 37°C, and 5% CO_2_ for 15 min. The cells were then observed under a laser confocal microscope (LSM 880, Carl Zeiss Meditec, Jena, Germany), and the numbers of red and green cardiomyocytes in each group were counted.

### ROS Detection

According to the instructions of the kit, H9c2 cells were inoculated into a six-well-plate. After culturing according to the above methods, the cells were collected and loaded with probes. After incubation in a cell incubator at 37°C for 20 min, the dichlorodihydrofluorescein diacetate (DCFH-DA) that did not enter the cells was removed with serum-free cell culture medium and analyzed by flow cytometry (Beckman, Suzhou, China).

### Annexin V-FITC/PI Detection

H9c2 cells were inoculated into six-well-plates. After treatment according to the experimental scheme, apoptosis was detected according to the operational instructions of the Annexin V-FITC/PI kit. The cells were digested with 0.25% trypsin, washed with PBS, and centrifuged. The cells were then resuspended in 200 μL buffer, 5 μL Annexin V-FITC, and 5 μL PI were added. After gentle mixing and incubation in the for 15 min, detection was carried out within 1 h using flow cytometry.

### Intracellular Western Blotting Analysis

Western blotting was used to detect the expressions of Sirt3, cytochrome C, Bax, and Bcl2. H9c2 cardiomyocytes were inoculated into a 25-cm^2^ culture flask at a density of 6 × 10^6^ cells/mL. After culturing the cells according to the method in Section Cell Culture and Hypoxia Condition, the cells were collected and washed twice with precooled PBS. The residual PBS was absorbed and discarded, and the cells were lysed on ice. The supernatant was collected by centrifugation at 4°C, and total protein was extracted using a total protein extraction kit. The total protein was quantified using a BCA kit. According to the method in Section Western Blotting Analysis, western blotting analysis was performed.

### Mitochondrial Western Blotting Analysis

Western blotting was used to detect the expressions of Drp1 and Opa1. H9c2 cardiomyocytes were inoculated into a 25-cm^2^ culture bottles at a density of 6 × 10^6^ cells/mL. The cells were collected after culturing according to the method specified in Section Cell Culture and Hypoxia Condition. After adding mitochondrial separation reagent and centrifuging at 4°C, the supernatant was carefully removed, and the isolated cell mitochondria were precipitated. According to the method in Section Western Blotting Analysis, western blotting analysis was performed. The VDAC1 protein level was used as a loading control.

### Statistical Analysis

All data were analyzed by SPSS 25 (IBM Corp., Armonk, NY, USA). All data are expressed as mean ± standard deviation (SD). All data conformed to normality using the Shapiro–Wilk test. One-way analysis of variance was used to analyze differences between groups with *P* < 0.05 indicating statistical significance.

## Results

### AS-IV Improved Cardiac Function and Reversed Left Ventricular Remodeling in the MI Model

**Figure 1:**
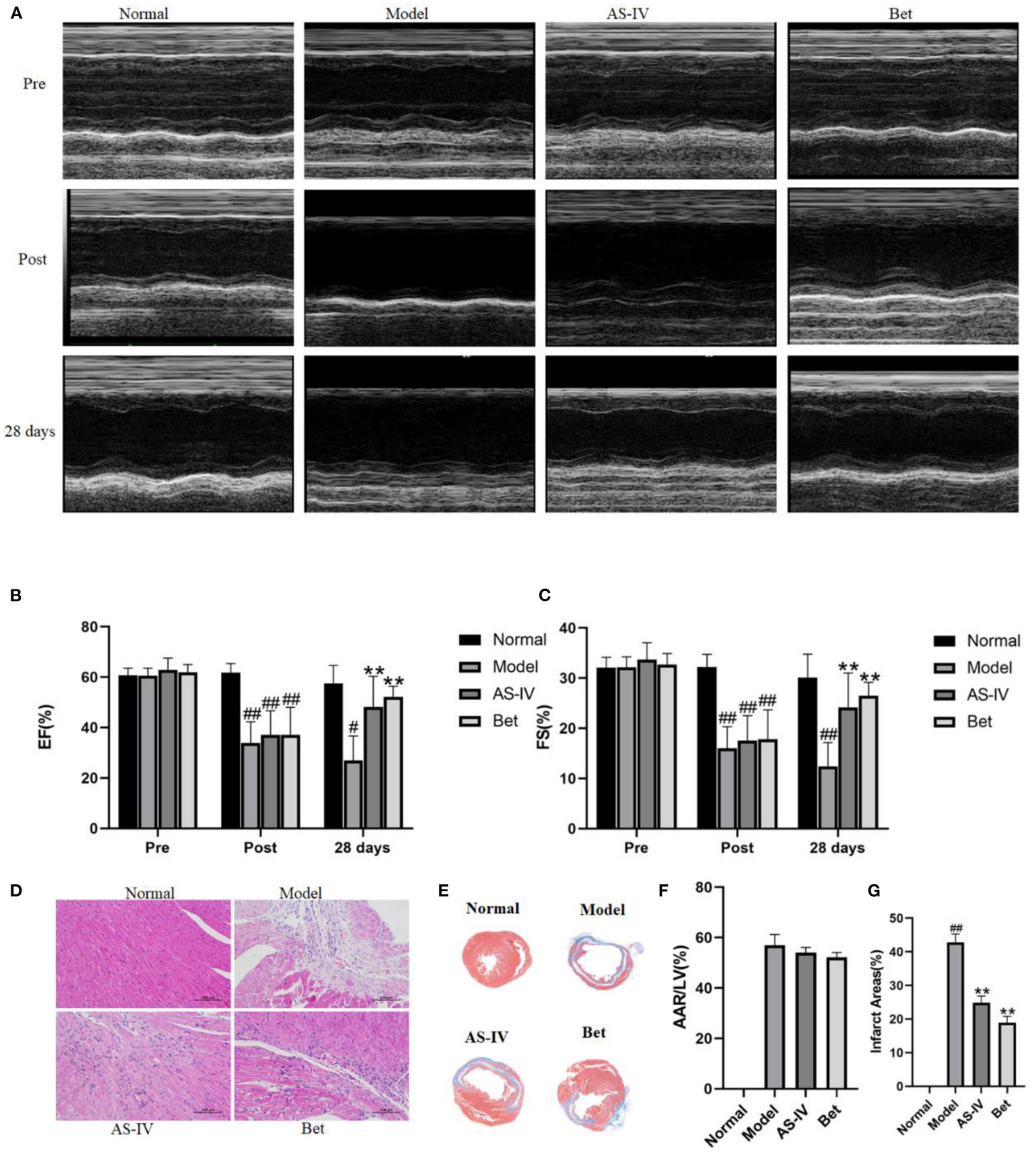
AS-IV protects the myocardium from ischemia-reperfusion injury. **(A)** M-mode echocardiographic images obtained before (pre), immediately after (post), and 28 days after the surgery to induce MI was completed. **(B,C)** Echocardiographic analysis of EF and FS. **(D)** H&E staining of the myocardium (scale bar = 100 μm). **(E)** Representative photograph of Masson staining. **(F)** Statistical analysis of area at risk/left ventricular (AAR/LV). **(G)** Statistical analysis of myocardial fibrosis. *n* = 4; ^#^*P* < 0.05, ^##^*P* < 0.01 vs. the normal group; **P < 0.01 vs. the model group.

Compared with the normal group, EF and FS were significantly decreased in the MI model group, indicating that MI seriously damaged the cardiac structure and function. AS-IV treatment increased EF and FS, demonstrating that were AS-IV had a significant protective effect on cardiac insufficiency and structural changes (Figures 1A–C). But the heart function of mice was weak, other echocardiography parameters did not reflect the difference of cardiac function in mice (Table 1). The mice in the normal group exhibited normal myocardial morphology with purplish red nuclei and light red cytoplasm. In the model group, myocardial vacuolization was obvious, collagen tissue was purple blue, myocardial fibrosis was obvious. In the AS-IV treatment group, we observed unclear cell boundaries, a small amount of neutrophil infiltration, cardiomyocytes swelling and degeneration, loose cytoplasm, and mild myocardial fibrosis. In the positive control group, the cardiomyocytes were swollen and degenerated, the cell boundary was unclear, the cytoplasm was loose and inconsistent, and mild myocardial fibrosis were observed (Figure 1D). Based on Masson staining, showed myocardium in the normal group was bright red, and the cytoplasmic was uniform. Interstitial collagen deposition was observed in the myocardial tissue of the model group. The collagen density of myocardial tissue in the AS-IV group and positive control groups were decreased significantly compared to the model group, while the collagen deposition was significantly improved. The AAR/LV ratio was not significantly different among the model group, the AS-IV group, and the Bet group, which suggested that the same ischemic area was present in the three groups. The infract areas in the model group was 42.7%. Treatment with AS-IV decreased the infract areas significantly to 24.9%. These results indicate that AS-IV can reduce myocardial fibrosis (Figures 1E–G).

**Table 1:** Echocardiographic parameters in different groups 4 weeks after treatment ($\bar{x}\;\pm$ s) (*n* = 4).

**Group**	**EF (%)**	**FS (%)**	**LVIDd (mm)**	**LVIDs (mm)**	**IVSd (mm)**	**IVSs (mm)**
Normal	59.102 ± 6.705	31.087 ± 4.533	0.707 ± 0.134	1.074 ± 0.150	3.954 ± 0.284	2.997 ± 0.235
Model	26.156 ± 11.113^##^	12.020 ± 5.394^##^	0.701 ± 0.326	0.800 ± 0.388	4.040 ± 1.218	3.597 ± 1.372
AS-IV	53.339 ± 4.018^**^	26.943 ± 2.447^**^	0.772 ± 0.094	1.197 ± 0.231	3.718 ± 0.130	2.726 ± 0.205
Bet	54.186 ± 5.193^**^	27.811 ± 3.472^**^	0.823 ± 0.188	1.227 ± 0.123	4.178 ± 0.335	3.012 ± 0.215

*EF, ejection fraction; FS, fractional shortening; LVIDd, left ventricular end diastolic internal diameter; LVIDs, left ventricular internal dimension in systole; IVSd and IVSs, interventricular septum thickness in diastole and systole, respectively.*

## *P < 0.01 vs. the normal group;*

** *P < 0.01 vs. the model group*.

### AS-IV Promoted Angiogenesis in MI Model Mice

**Figure 2:**
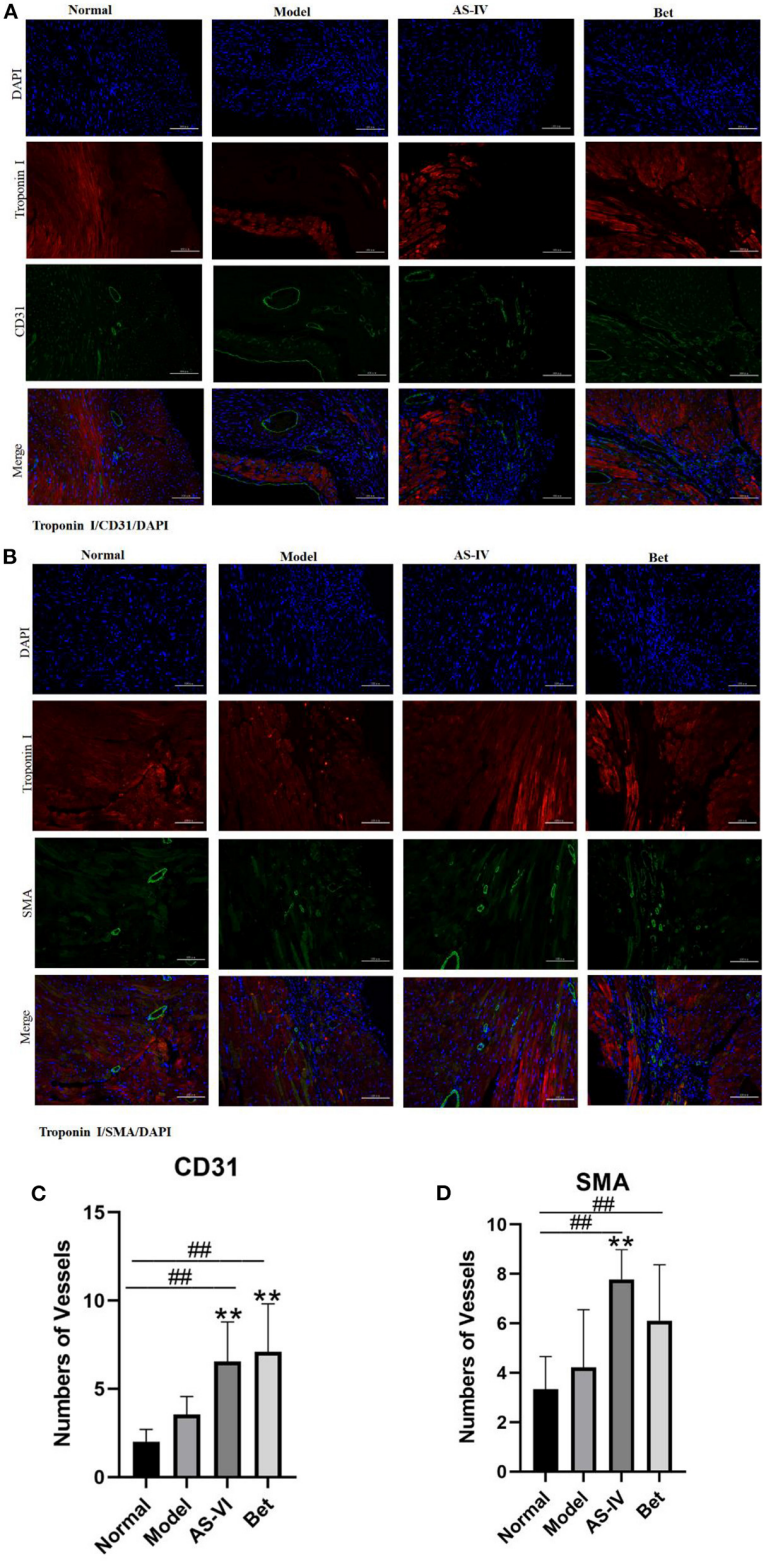
AS-IV promoted angiogenesis in infarcted mouse heart. **(A,C)** CD31 and SMA expression to identify endothelial cells and smooth-muscle cells. **(B,D)** Vessels were quantified as the number of CD31-positive structures and SMA-positive structures (scale bar = 100 μm). *n* = 4; ^##^*P* < 0.01 vs. the normal group; **P < 0.01 vs. the model group.

CD31 and SMA were used to indicate capillary density and arteriole formation, respectively. Both capillary density and arteriole density enhanced after MI, and CD31 staining positive cells were significantly increased in the AS-IV group compared with the model group. Similarly, the formation of small and medium arteries in the AS-IV group was significantly increased compared with the model group. These results indicate that AS-IV can increase capillaries and arterioles after operation for MI (Figures 2A–D).

### AS-IV Improved Mitochondrial Morphology

**Figure 3:**
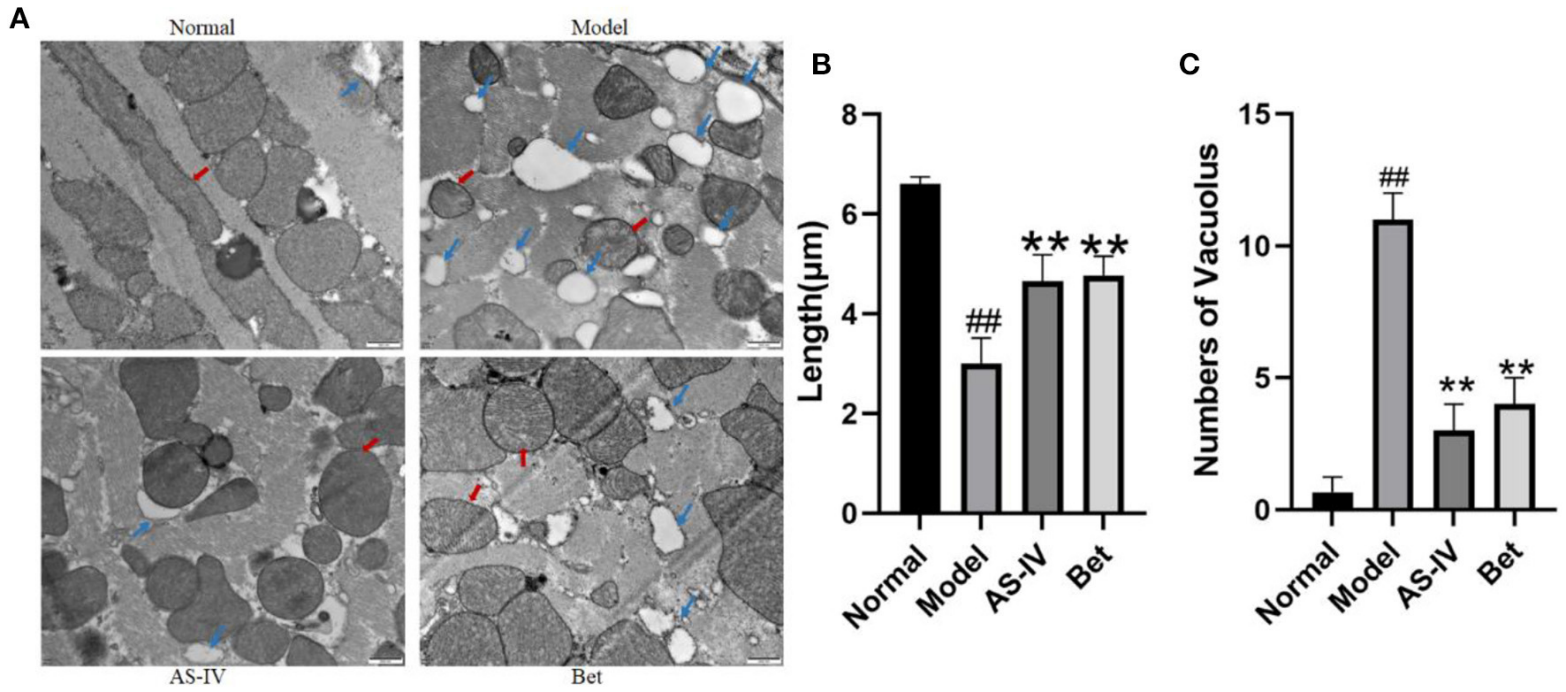
Changes in the mitochondrial structure in the myocardial tissue of mice. **(A)** Changes in the microvascular ultrastructure of myocardial tissue from the infarct border zone under transmission electron microscopy (scale bar = 500 nm). **(B)** The mitochondrial length was measured at least four fields for each group. **(C)** Vacuolar structure of myocardial mitochondria from the infarct border zone (scale bar = 500 nm). Red arrows point to the ultrastructure of mitochondria, and blue arrows point to the vacuolization in mitochondria. *n* = 4; ^##^*P* < 0.01 vs. the normal group; **P < 0.01 vs. the model group.

In the normal group, the ultrastructure of myocardial mitochondria was clear, the mitochondrial bilayer membrane was clear, and the sarcomeres showed ordered arrangement; In contrast, the ultrastructure of myocardial mitochondria in the model group showed obvious vacuolization and partial double-layer membrane fusion. In the AS-IV group, the myocardial mitochondria were slightly vacuolated, and the sarcomere arrangement was slightly disordered. In the positive control treatment group, the myocardial mitochondria were slightly vacuolized and exhibited edema (Figures 3A,C). The mitochondrial length was significantly shorter after exposure to hypoxia than that under normoxia, whereas the mitochondrial length was longer in the AS-IV group than that in the model group (Figure 3B).

### AS-IV Reversed MI *via* Sirt3/Drp1 Pathway

**Figure 4:**
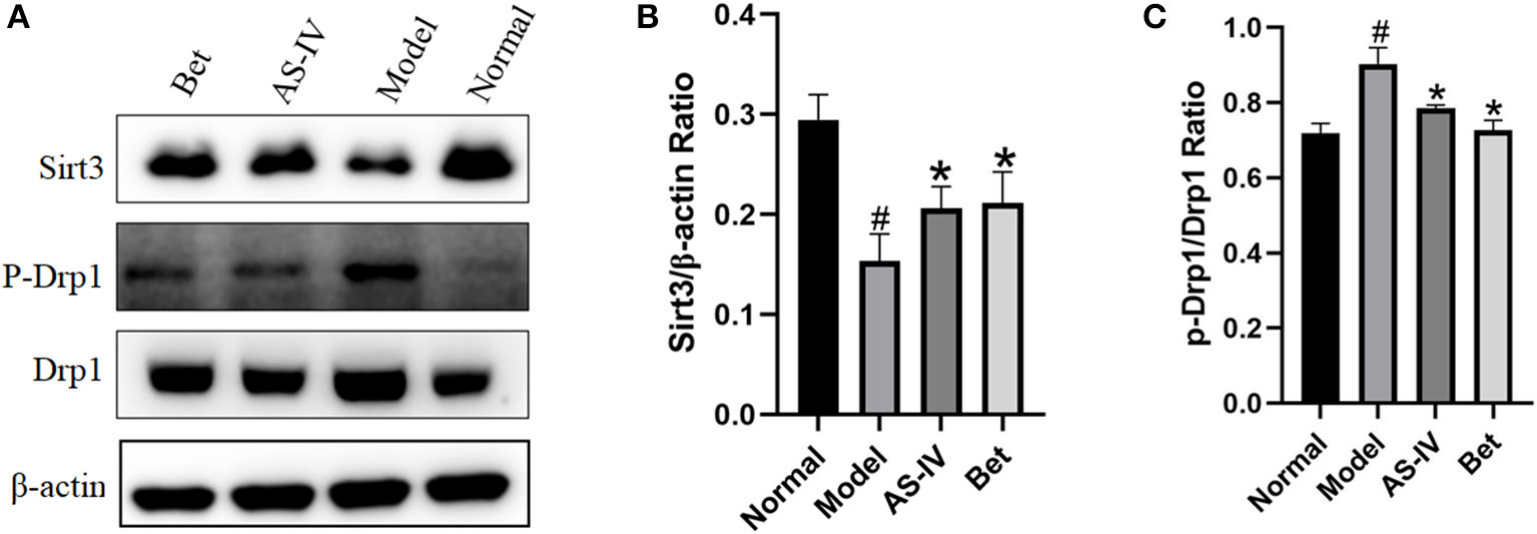
AS-IV reversed MI *via* Sirt3/Drp1 pathway. **(A–C)** Sirt3, p-Drp1, and Drp1 protein levels in the normal, model, and AS-IV groups evaluated by western blotting. The β-actin levels were evaluated to confirm equal loading. *n* = 3; ^#^*P* < 0.05 vs. the normal group; *P < 0.05 vs. the model group.

Compared with the normal group, the expression of Sirt3 in the model group was down-regulated, and the expression of p-Drp1/Drp1 was up-regulated. Compared with the model group, the expression of Sirt3 in the AS-IV group was up-regulated, and the expression of p-Drp1/Drp1 was down-regulated. These results indicate that AS-IV effectively activate the Sirt3/Drp1 signaling pathway induced by hypoxia (Figures 4A–C).

### AS-IV Inhibited Hypoxia-Induced Apoptosis in H9c2 Cells

**Figure 5:**
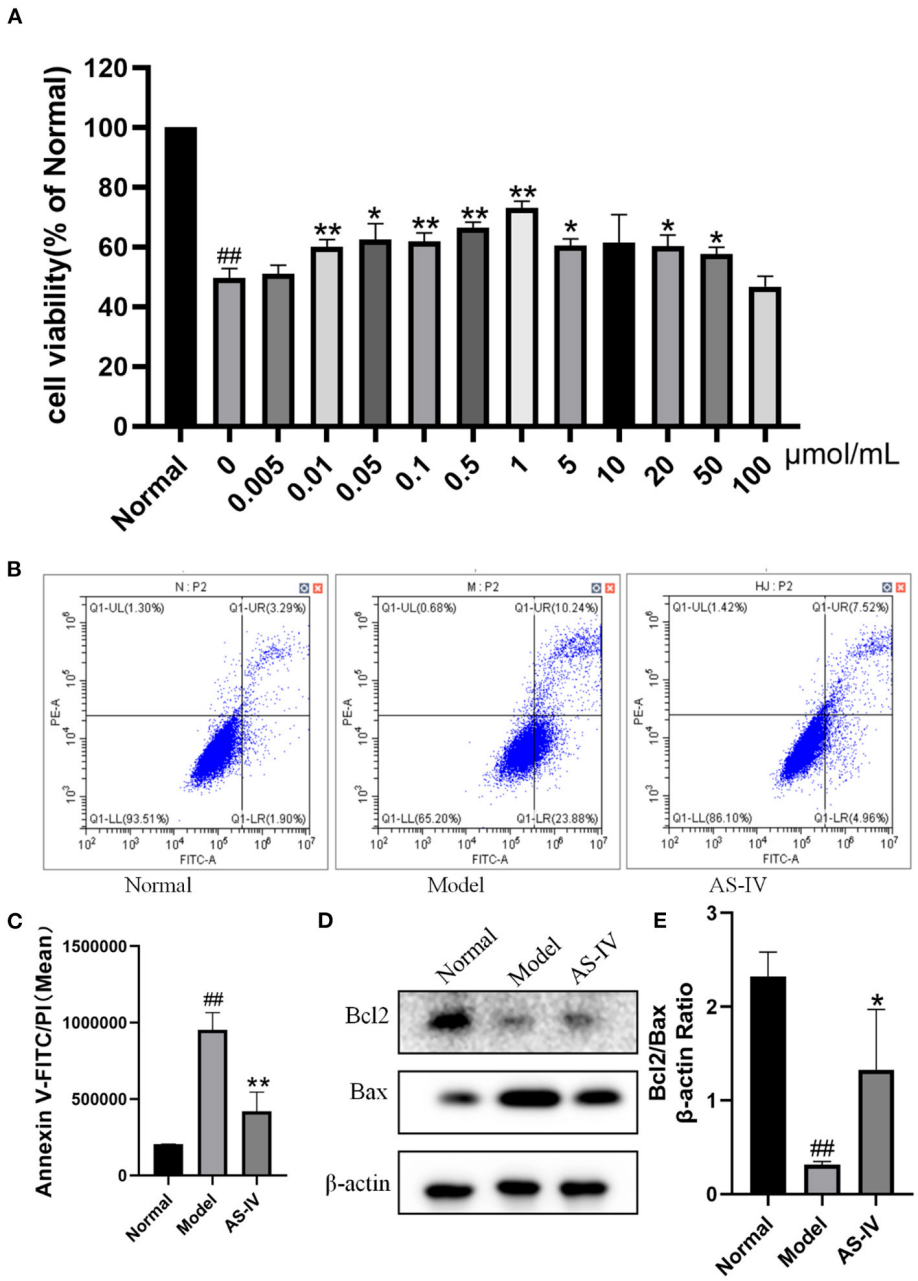
Effects of AS-IV on H9c2 cells after hypoxia. **(A)** Effects of different concentrations of AS-IV on the survival rate of hypoxia injured H9c2 cardiomyocytes determined by CCK-8 assay. **(B,C)** Quantification of Annexin V-FITC/PI in the normal, model, and AS-IV groups. **(D,E)** Bcl2 and Bax protein levels in the normal, model, and AS-IV groups evaluated by western blotting. The β-actin levels were also evaluated to confirm equal loading. *n* = 3; ^##^*P* < 0.01 vs. the normal group; *P < 0.05, **P < 0.01 vs. the model group.

Hypoxia treatment significantly decreased the cell viability, whereas treating the MI mice with AS-IV significantly increased cell viability. Among the tested AS-IV concentrations, 1 μM had the most significant protective effect and the cell viability reached 75%. One micrometer was selected for further experiments (Figure 5A). The Annexin V-FITC/PI response is well-known in apoptosis. The apoptosis rate was lower in the AS-IV group (14%) than that in the model group (35%). Bcl2 as anti-apoptotic protein, and Bax a pro-apoptotic protein, play important roles in regulating apoptosis. Compared with the control H9c2 cells, the model cells exhibited significantly decreased Bcl2 expression along with significantly increased Bax expression, and these changes were improved by AS-IV treatment (Figures 5B,C). These results are consistent with the Annexin V-FITC/PI analysis (Figures 5D,E).

### AS-IV Maintained Mitochondrial Integrity After Hypoxia

**Figure 6:**
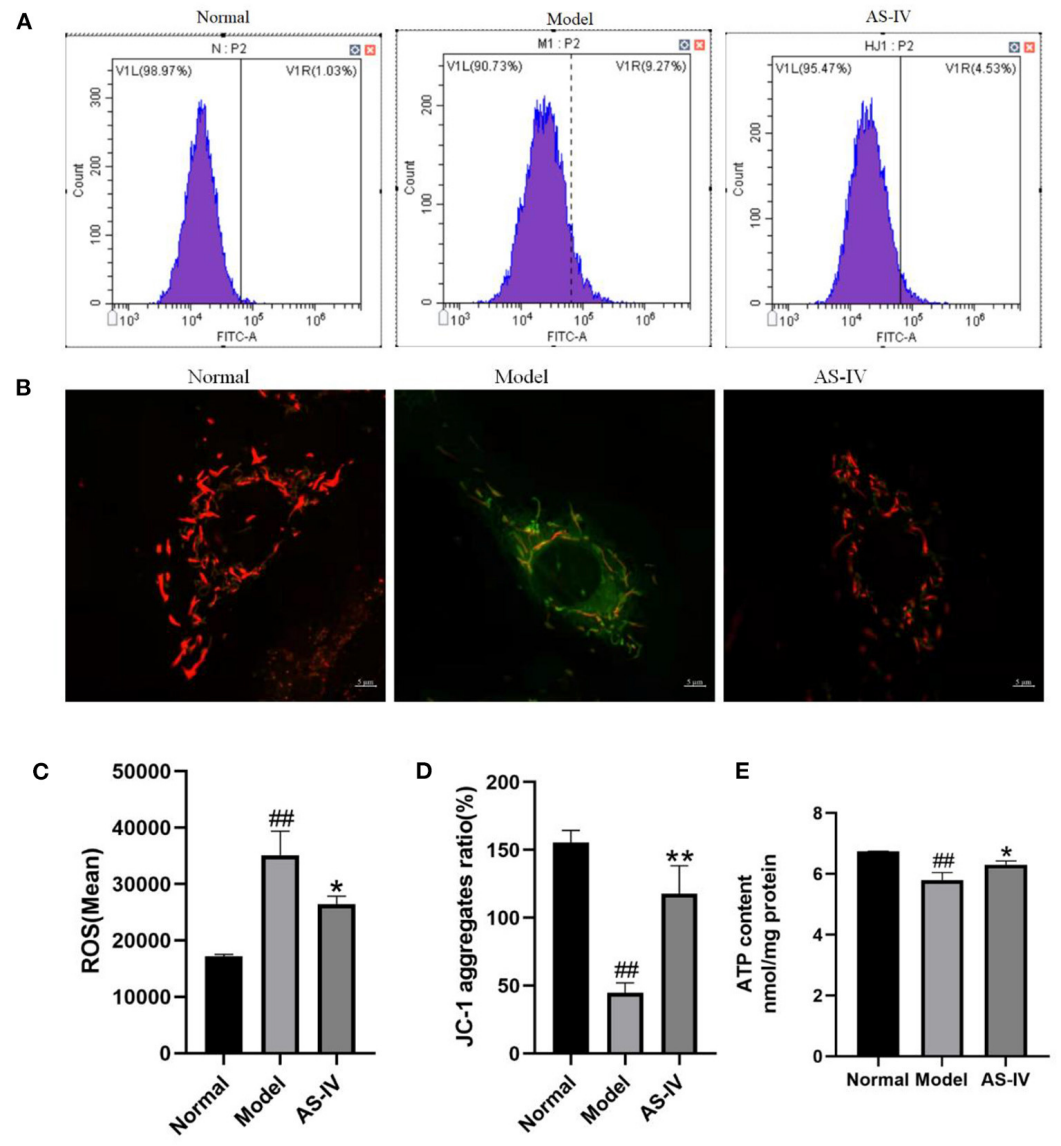
AS-IV mediated mitochondrial integrity and inhibited cell apoptosis. **(A,C)** ROS contents estimated by ROS assay. **(B,D)** ΔΨM values assessed *via* JC-1 staining (scale bar = 5 μm). **(E)** ATP levels measured using an ATP detection kit. *n* = 3; ^##^*P* < 0.01 vs. the normal group; *P < 0.05, **P < 0.01 vs. the model group.

DCFH-DA, which has membrane permeability but not fluorescence, was used as a probe for active oxygen detection. Once DCFH-DA enters cells, it is hydrolyzed by cellular esterase to 2′,7′-dichlorodihydrofluorescein (DCFH). In the presence of ROS, DCFH can be rapidly oxidized to 2′,7′-dichlorofluorescein (DCF), which cannot penetrate the cell membrane. The mean fluorescence intensity of DCF is directly proportional to the level of ROS in cells (41). In this study, the intracellular ROS level was significantly higher in the model group than in the control group. Compared with the model group, AS-IV treatment significantly reduced the level of ROS (Figures 6A,C). The levels of ΔΨM and ATP indicated mitochondrial dysfunction and apoptosis. To evaluate the protective effect of AS-IV against mitochondrial membrane damage induced by hypoxia, the ΔΨM and ATP levels were determined as indicators of the mitochondrial function of H9c2 cells (42, 43). The cells in the normal group showed red aggregation and normal hyperpolarized membrane potential. After hypoxia, the cells in the model group showed obvious green JC-1 monomers (Figures 6B,D), decreased mitochondrial membrane potential (MMP), and decreased ATP level. However, in the AS-IV group, the ATP level increased, the decrease in MMP was reduced. Light red and orange fluorescence was observed. These results suggest that AS-IV significantly ameliorated the hypoxia-induced decreases in MMP and ATP level (Figure 6E).

### The Benefits of AS-IV in Mitochondria Are Related to the Expressions of Sirt3 and Drp1

**Figure 7:**
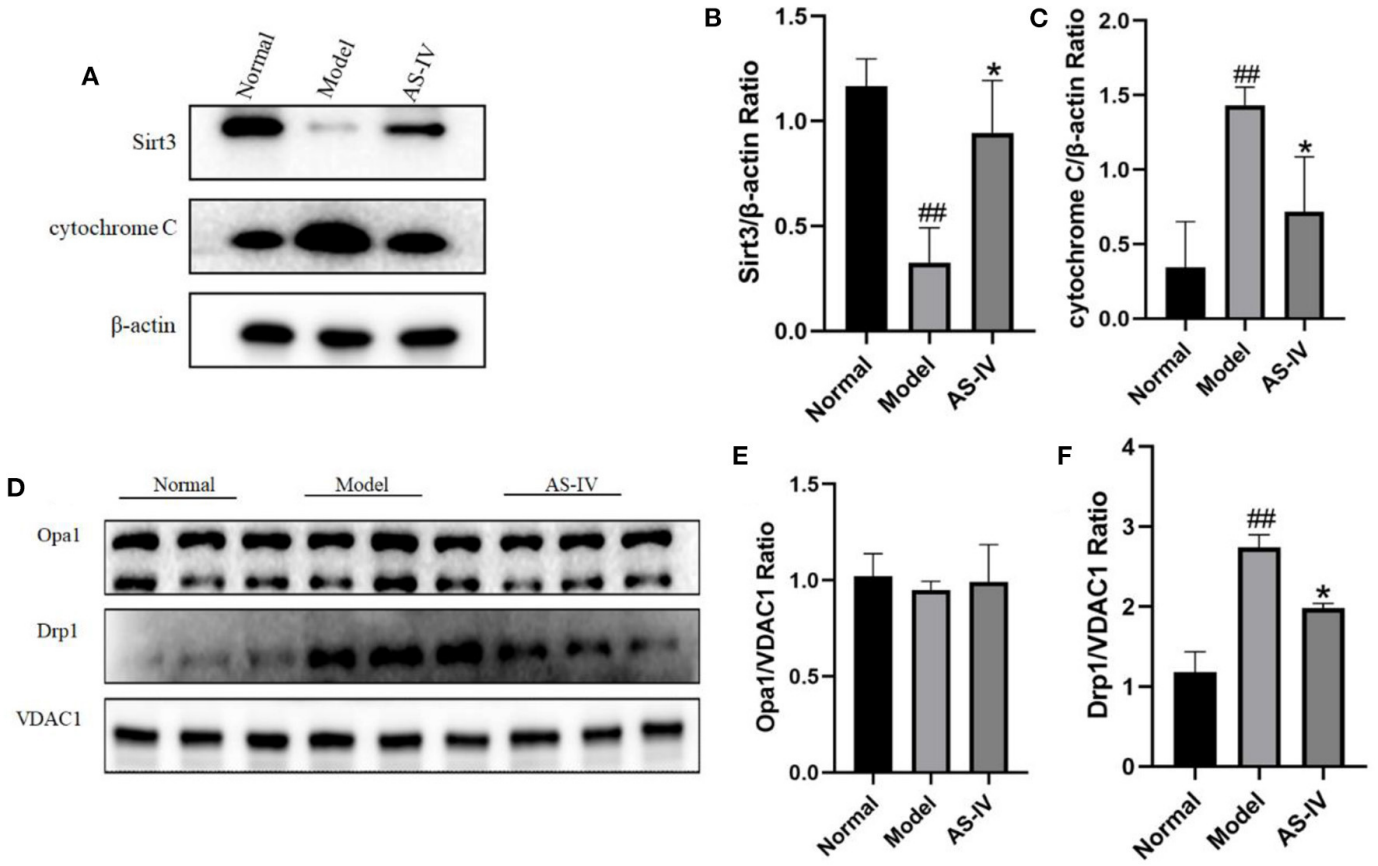
AS-IV maintained mitochondrial integrity and inhibited cell apoptosis through the Sirt3/Drp1 pathway. **(A–C)** Expressions of Sirt3 and cytochrome C in the normal, model, and AS-IV groups determined *via* western blotting. The β-actin levels were also evaluated to confirm equal loading. **(D–F)** Levels Opa1 and Drp1 in the normal, model, and AS-IV groups. The VDAC1 levels were also evaluated to confirm equal loading. *n* = 3; ^##^*P* < 0.01 vs. the normal group; **P < 0.01 vs. the model group.

Sirt3 and the mitochondria related proteins Opa1 and Drp1 are involved in the regulation of mitochondrial function along with cardiomyocyte apoptosis after hypoxia. After hypoxia, the expression of Sirt3 decreased, the expression of Drp1 increased and the level of cytochrome C increased; in contrast, these changes were not observed in the AS-IV group (Figures 7A–D,F). However, the expression of Opa1 did not change significantly (Figure 7E).

### The Absence of Sirt3 Attenuates the Protective Effects of AS-IV on H9c2 Cells After Hypoxia

**Figure 8:**
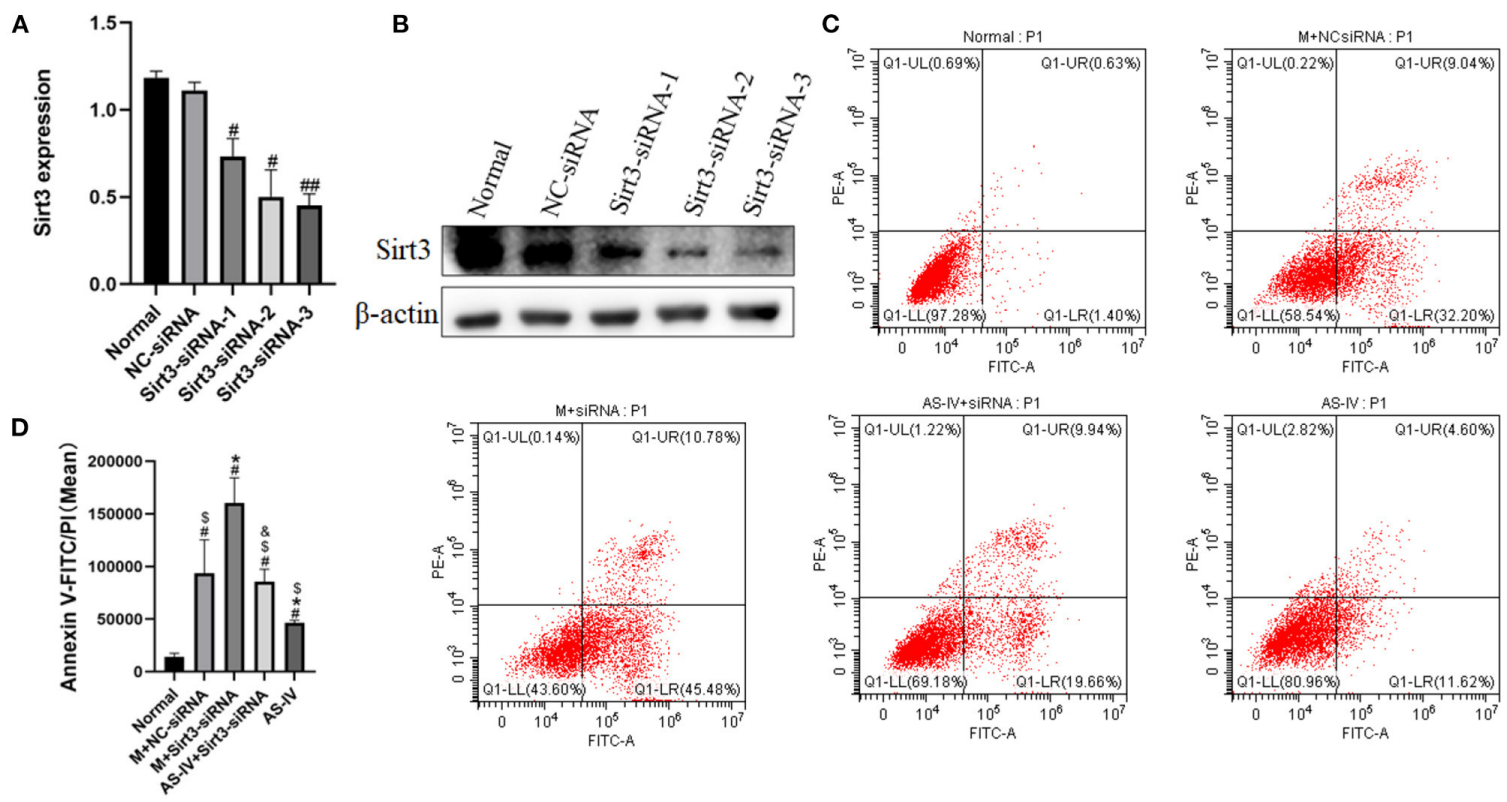
The absence of Sirt3 attenuates the protective effects of AS-IV on H9c2 cells after hypoxia. **(A,B)** The efficiency of Sirt3 silencing was evaluated *via* western blotting. **(C,D)** Quantification of Annexin V-FITC/PI was used to analyze cell apoptosis. *n* = 3; ^#^*P* < 0.05, ^##^*P* < 0.01 vs. the normal group; *P < 0.05 vs. the M+NC-siRNA group; ^$^*P* < 0.05 vs. the M+Sirt3-siRNA group; ^&^*P* < 0.05 vs. the AS-IV group.

To figure out the effects of AS-IV and Sirt3 on hypoxia-induced cells, we synthesized three siRNAs targeting Sirt3 and then transfected them into H9c2 cells. The expression of Sirt3 was assessed *via* western blotting (Figures 8A,B). We observed that Sirt3-siRNA-3 exhibited the best interference efficiency and labeled the treated cells as the Sirt3-siRNA group. Subsequently, apoptosis was evaluated after AS-IV treatment and Sirt3 silencing. The results showed that Sirt3 silencing abolished the ability of AS-IV to decrease apoptosis after hypoxia (Figures 8C,D), suggesting that Sirt3 is necessary for AS-IV-mediated cardio protection.

### AS-IV Regulates Mitochondrial Dynamics by Enhancing Sirt3 Expression and Activity

**Figure 9:**
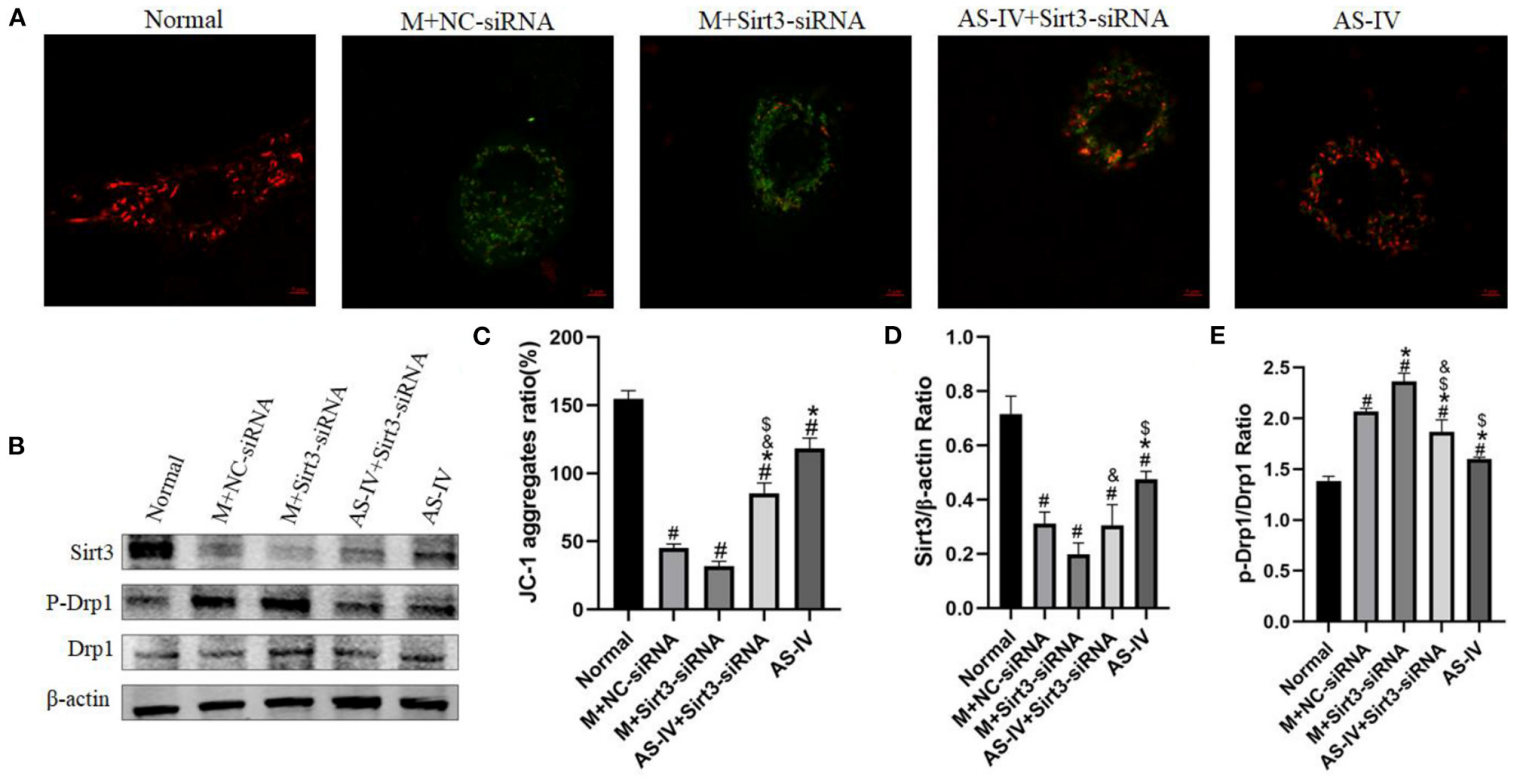
AS-IV regulates mitochondrial dynamics by enhancing Sirt3 expression and activity. **(A,C)** MMP was analyzed by JC-1 staining and expressed as the ratio of red/green fluorescence intensity. **(B,D,E)** Expressions of Sirt3, p-Drp1, and Drp1 were determined *via* western blotting. *n* = 3; ^#^*P* < 0.05 vs. the normal group; *P < 0.05 vs. the M+NC-siRNA group; ^$^*P* < 0.05 vs. the M+Sirt3-siRNA group; ^&^*P* < 0.05 vs. the AS-IV group.

We wished to ascertain if Sirt3 is necessary for AS-IV to maintain mitochondrial homeostasis. Hence, we assessed the ΔΨM using JC-1 staining and Sirt3 expression in H9C2 cells. The results showed that compared to the normal group, hypoxia decreased the MMP, and AS-IV increased the MMP, an effect of that was eliminated by Sirt3 silencing (Figures 9A–D). We demonstrated that the mitochondrial fission related protein p-Drp1/Drp1 was up-regulated in response to hypoxia (Figure 9E). AS-IV treatment reduced the levels of p-Drp1/Drp1 that was achieved by increasing Sirt3 activity. These findings suggest that AS-IV maintains mitochondrial homeostasis.

## Discussion

Li (34) found that AS-IV at dose of 10 mg/kg improved cardiac function in mice with MI. So, according to the literature, we selected the dose of 10 mg/kg for AS-IV in treating MI. In the present study, we confirmed that the permanent ligation of the left anterior descending coronary artery can cause pathological damages including fibrosis, interstitial edema, and inflammatory cell infiltration in mouse myocardial tissue along with decreases in EF and FS. Treatment with AS-IV significantly ameliorated these pathological abnormalities, consistent with previous results (25, 44), indicating that AS-IV can prevent and treat MI. We observed serious mitochondrial swelling, cristae disorder, and other structural damages in mouse cardiomyocytes after MI, suggesting that the mitochondrial structure was broken after MI, which would eventually lead to apoptosis and loss of function. AS-IV treatment significantly improved the ultrastructure of mitochondria in the myocardial ischemic area and increased the ATP content and membrane potential.

To evaluate the mechanism by which AS-IV inhibits cardiomyocyte apoptosis and reduces cardiomyocyte injury after hypoxia, indexes related to of mitochondrial structure were analyzed. The Bcl2 of family pro-apoptotic proteins plays a key role in the regulation of mitochondrial apoptosis (45). Bax can promote apoptosis, inhibit the effect of its homolog Bcl2, reduce the level of cytochrome C, and accelerate apoptosis (46, 47). Bcl2 inhibits apoptosis induced by many factors and affects the apoptosis rate of myocardial cells in MI (48). Cell viability was estimated by CCK-8 assay. Among the tested AS-IV concentrations, 1 μM had the most significant protective effect and 1 μM was selected for further experiments. In the current study, AS-IV treatment significantly reduced the expression of Bax and increased the expression of Bcl2 compared to the model group, suggesting that AS-IV can help inhibit cardiomyocyte apoptosis and protect cardiac function.

Sirt3 exerts anti-apoptotic effects by directly binding and deacetylating Ku70, promoting the interaction of Ku70 with the pro-apoptotic protein Bax, and impeding the translocation of Bax to mitochondria (49). As an important mitotic protein, Drp1 affects mitochondrial morphology and participates in the regulation of apoptosis (50). Drp1 stimulates Bax oligomerization initiated by tBid by promoting the half-fusion of cardiolipin-containing membranes and releasing cytochrome C through the membrane half-fusion intermediate formed during mitochondrial division, thereby initiating apoptosis (51, 52). Liu et al. (53) found that mitochondrial fission increased after MI, leading to mitochondrial oxidative stress, metabolic disorders, and reduced membrane potential, thereby resulting in cardiomyocyte apoptosis. The overexpression of Sirt3 can reduce mitochondrial fission by normalizing the AMPK-Drp1 pathway. The inhibitory effect of Sirt3 on mitochondrial division can be eliminated by increasing the activity of Drp1. Opa1 is a key regulatory protein of mitochondrial inner membrane fusion. Opa1 plays an important role in mitochondrial ridge formation and morphological maintenance. Opa1 can oligomerize to regulate mitochondrial ridge remodeling during apoptosis (54, 55). In addition, Opa1 deletion can expand the mitochondrial ridge area (56). However, in the current study western blot analysis indicated that the expression of Opa1 did not change after hypoxia. Thus, the role of Opa1 requires further research.

The present study has some limitations. We did not use Drp1 inhibitors to confirm the role of Drp1 deficiency in H9c2 cells exposed to hypoxia. The metabolic data and mitochondrial respiration were not tested in this manuscript. In this manuscript, only mitochondrial division and related proteins were detected, but membrane associated membrane integrity was not detected. They will be tested in subsequent experiments.

In conclusion, we have demonstrated that AS-IV can ameliorate the negative effects of MI in mice. The mechanism of these effects is related to the up-regulation of Sirt3 and Drp1 by AS-IV. These findings provide a new strategy for cardiac protection using AS-IV.

## Data Availability Statement

The original contributions presented in the study are included in the article/Supplementary Material, further inquiries can be directed to the corresponding author.

## Ethics Statement

The animal research protocol was approved by the Institutional Animal Care and Use Committee of the Laboratory Animal Research Center of Zhejiang Chinese Medical [License No. SYXK (Zhe)2018–0012].

## Author Contributions

HW, JY, and YH conceived the idea and designed the study. WZ and LZ performed the experiments. HZ wrote the manuscript. CS and CL participated in the data acquisition and statistical analysis. All authors contributed to the article and approved the submitted version.

## Funding

This work was supported by the grants from National Key R&D Program of China (Nos. 2019YFC1708600, 2019YFC1708604), National Natural Science Foundation of China (No. 81973560), and Key Laboratory of TCM Encephalopathy of Zhejiang Province (No. 2020E10012).

## Conflict of Interest

The authors declare that the research was conducted in the absence of any commercial or financial relationships that could be construed as a potential conflict of interest.

## Publisher's Note

All claims expressed in this article are solely those of the authors and do not necessarily represent those of their affiliated organizations, or those of the publisher, the editors and the reviewers. Any product that may be evaluated in this article, or claim that may be made by its manufacturer, is not guaranteed or endorsed by the publisher.

## Abbreviations

AS-IV: astragaloside IV
Sirt3: silent information regulator 3
MI: myocardial infarction
Opa1: optic atrophy protein-1
Drp1: dynamin-related protein 1
α-SMA: α-smooth muscle actin
Bet: Betloc
EF: ejection fraction
FS: fractional shortening
H&E: hematoxylin and Eosin
PBS: phosphate-buffered saline
BSA: bovine serum albumin
DMEM: Dulbecco's modified eagle medium
FBS: fetal bovine serum
DCFH-DA: dichlorodihydrofluorescein diacetate
DCFH, 2′: 7′-dichlorodihydrofluorescein
DCF, 2′: 7′-dichlorofluorescein
MMP: mitochondrial membrane potential.
